# Silanization of Chitosan and Hydrogel Preparation for Skeletal Tissue Engineering

**DOI:** 10.3390/polym12122823

**Published:** 2020-11-27

**Authors:** Gildas Réthoré, Cécile Boyer, Kouakou Kouadio, Amadou Toure, Julie Lesoeur, Boris Halgand, Fabienne Jordana, Jérôme Guicheux, Pierre Weiss

**Affiliations:** 1 Dental Faculty, Université de Nantes, UMR 1229, RMeS, Regenerative Medicine and Skeleton, INSERM, ONIRIS, F-44042 Nantes, France; gildas.rethore@univ-nantes.fr (G.R.); cecile.boyer@univ-nantes.fr (C.B.); kouadiokouakou@yahoo.fr (K.K.); amad_toure@yahoo.fr (A.T.); julie.lesoeur@univ-nantes.fr (J.L.); boris.halgand@univ-nantes.fr (B.H.); fabienne.jordana@univ-nantes.fr (F.J.); jerome.guicheux@univ-nantes.fr (J.G.); 2 Institut National de la Santé et de la Recherche Médicale, Université de Nantes, UFR Odontologie, F-44042 Nantes, France; 3 CHU Nantes, PHU4 OTONN, F-44093 Nantes, France; 4 Department of Odontology, Faculty of Medicine, Pharmacy and Odontology, University Cheikh Anta DIOP, 12500 Dakar, Senegal

**Keywords:** cartilage, chitosan, hydrogel, tissue engineering, silane

## Abstract

Tissue engineering is a multidisciplinary field that relies on the development of customized biomaterial to support cell growth, differentiation and matrix production. Toward that goal, we designed the grafting of silane groups onto the chitosan backbone (Si-chito) for the preparation of in situ setting hydrogels in association with silanized hydroxypropyl methylcellulose (Si-HPMC). Once functionalized, the chitosan was characterized, and the presence of silane groups and its ability to gel were demonstrated by rheology that strongly suggests the presence of silane groups. Throughout physicochemical investigations, the Si-HPMC hydrogels containing Si-chito were found to be stiffer with an injection force unmodified. The presence of chitosan within the hydrogel has demonstrated a higher adhesion of the hydrogel onto the surface of tissues. The results of cell viability assays indicated that there was no cytotoxicity of Si-chito hydrogels in 2D and 3D culture of human SW1353 cells and human adipose stromal cells, respectively. Moreover, Si-chito allows the transplantation of human nasal chondrocytes in the subcutis of nude mice while maintaining their viability and extracellular matrix secretory activity. To conclude, Si-chito mixed with Si-HPMC is an injectable, self-setting and cytocompatible hydrogel able to support the in vitro and in vivo viability and activity of hASC.

## 1. Introduction

Tissue engineering is a multidisciplinary approach that incorporates biology, medicine and engineering [1]. As a field of study, the discipline of tissue engineering aims to understand the relationship between structure and function in cell and tissue and to develop biological substitutes that can repair or replace the dead or damaged tissues, organs and/or parts of the human body [2]. The success of this strategy lies in the synergy of three components: (i) cells for the tissue formation, (ii) biomaterials to act as scaffolds and (iii) bioactive molecules to guide the cells to form the desired tissue type [3,4,5].

Throughout the literature, several strategies have been developed to prepare the ideal scaffold [6,7,8]. Hydrogels are of particular interest in tissue engineering applications due to their hydrophilic character, nanoporous structure, high water content and often biocompatible nature [9,10,11,12,13].

Numerous biomaterials have been used for the fabrication of hydrogels, including natural materials derived from animals or plants (collagen, fibrin, hyaluronan and chitosan) and synthetic materials with a wide range of synthetic polymers [14]. Scaffolds composed of natural origin polymers are attractive owing to their biocompatibility, biodegradability, low toxicity, chronic inflammatory response and their biological characteristics and structural similarities with human tissues.

In the past few years, the authors have been developing a cellulose-based hydrogel (Si-HPMC). The silanization of HPMC with pendant alkoxysilane or silanolate groups allows reversible, pH-dependent, polycondensation by sol–gel route without any reactants that are often cytotoxic, thus allowing the injected hydrogel to fit and remain at the damaged area. The deficiencies of silated HPMC is that it is a low degradable hydrogel [15], and it has no interactions with tissues or cells [16]. This Si-HPMC hydrogel has already been demonstrated to be a convenient matrix for the three dimensional (3D) culture of hASC and hNC [9,17,18]. However, hydrogels suffer from several drawbacks such as low mechanical and biodegradable properties, weak adhesion capacity.

To overcome these problems, one of the candidates of interest as natural polymeric material for scaffold preparation would be chitosan (chitin-derived polymer) [19]. Chitin is the second abundant biopolymer on earth, following cellulose [20,21].

Chitosan, a deacetylated chitin, is a polymer that has free amine groups within its backbone chain, thus has the characteristics of a polymeric hydrogel owing to a high water absorption capacity [22]. Chitosan is also known to possess muco-adhesivity (biodegradability, antimicrobial activity [23,24] and low toxicity and immunogenicity, which are essential for scaffolds [25,26]. For these reasons, chitosan has found a tremendous variety of biomedical applications in recent years [27].

This paper deals with the functionalization of chitosan with silane groups towards the development of an injectable self-setting chitosan-based hydrogel. First of all, we reported the protocol and the efficacy of the functionalization of chitosan. Then, the formulation of the silated chitosan (Si-chito) was described. Following the preparation of the hydrogels, their rheological and mechanical characterizations were performed, and the cell viability in 2D and 3D was evaluated. Finally, the preclinical relevance of our Si-chito hydrogels was investigated in subcutaneous nude mouse cells.

## 2. Materials and Methods

### 2.1. Materials

The 4-(2-hydroxyethyl)-1-piperazine-ethanesulfonic acid (HEPES, H4034), (3-Isocyanatopropyl)triethoxysilane (ref 413,364) and the chitosan (75–85% deacetylated, Mw 50,000–190,000 Da, viscosity 20–300 cP, ref 448869) were purchased from Sigma Aldrich, Darmstadt, Germany. The NaCl (27,810.295) and the HCl (37 wt %, 20,252.335) were ordered from VWR, Radnor, PA, USA.

### 2.2. Si-HPMC and Si-Chito Synthesis

Silanized hydroxypropyl methylcellulose (Si-HPMC) and the acidic buffer solution (BS, pH 3.6) were prepared as previously described [28,29,30]. With 420 mL of 1-propanol, 1.9 L of n-heptane was stirred. To the mixture under stirring, 12 g of NaOH and 240 g of dry HPMC were added. The mixture was kept at room temperature for 50 min under a nitrogen bubbling. A 36 mL of 3-GPTMS, which was the group to be grafted, was added dropwise, and the temperature was increased until 85 °C in 35 min. The boiling was kept for 3.5 h. Heating was closed, and at 40 °C, 30 mL of glacial acetic acid was poured for the reaction neutralization. After 30 min, the mixture was filtered using a Buchner funnel. The powder was washed successively four times with 3 L of an acetone/water mixture (85:15 *v/v*) to eliminate unreacted GPTMS, and HPMC-Si powder was dried at 37 °C. Silanized chitosan (Si-chito) was synthesized as follows: 100 mL of chitosan (1% wt/v, HCl, pH 3) was poured in a round-bottomed flask and from 1 to 2.5 eq (1.55 mL to 3.875 mL) of (3-Isocyanatopropyl)triethoxysilane (IPTS) was added. The mixture was then stirred from 1 h to 5 h at room temperature. The synthesis conditions are listed in Table 1.

SEM-EDX monitoring was used to determine the efficiency of the grafting, and coupled plasma atomic emission spectroscopy (ICP-ES) was used to determine the amount of grafted silane (for the selected condition), and the percentage (*wt*/*wt*) was measured as 1.5%.

### 2.3. Hydrogel Preparation

Si-HPMC polymer (3 wt %) was dissolved in 0.2 M NaOH aqueous solution; then Si-chito (from 0 to 3 wt %) was dissolved in 3 wt % Si-HPMC aqueous solution (0.2 M NaOH). Finally, two dialyzes with a molecular weight cutoff at 6–8 kDa (Spectra/Por^®^1 standard RC tubbing, 20.4 mm-diameter) were performed in 0.09 M NaOHaq. The dialyzes eliminate the non-grafted silane derivatives used for siloxane grafting onto the HPMC and Chitosan. The hydrogel precursor solution was then obtained by mixing 1 volume of the above Si-HPMC/Si-chito basic solution contained in one Luer-lock syringe, with 0.5 volume of acidic BS in another Luer-lock syringe, by the interconnection of both syringes; the final pH is 7.4. This precursor mixture was still injectable at room temperature during 30–40 min until the gel point was reached. A 0 wt % Si-chito hydrogel consists of 98 wt % of water and 2 wt % of Si-HPMC polymer, and a 2 wt % Si-chito hydrogel consists of 96% wt % of water and 2 wt % of Si-HPMC and 2 wt % of Si-chito polymers.

Prior to any experiments, Si-HPMC, Si-HPMC/Si-chito basic solution and acidic buffer solutions (ABS) were autoclaved at 120 °C for 20 min with an Alphaklave 23.

### 2.4. Viscosity Measurements

Rheological measurements were performed using the Haake MARS rheometer (ThermoHaake^®^, Dreieich, Germany) with a titanium cone-plate geometry (60 mm-diameter, 1° cone angle, 52 µm gap). Steady shear tests were carried out at 25 °C on different Si-HPMC and Si-HPMC/Si-chito solutions (2 and 3 wt %). The operating shear rate ranged from 0.1 to 9000 s^−1^. Different flow curves were fitted and extrapolated to lower shear rates by the Cross equation [31].

### 2.5. Syringeability

Injectability properties were investigated using a compression test device (TAHDplus) with a 5 kg load cell utilized for the measurements at an injectability rate of 17 sec per mL through an 18G needle. A syringe containing 1 mL of hydrogel precursor, after mixing Si-HPMC or Si-chito with the acidic buffer, was set, and the injection force was measured.

### 2.6. Gel Point Measurements

The gel points were obtained on HAAKE RheoStress RS300 rheometer (Thermo Scientific, Dreieich, Germany) using cone/plate geometry (C60/1°Ti, titanium 1° angle and 60 mm of diameter). Liquid hydrogel precursor solutions were injected onto the plate immediately after mixing, and the measurements started 1–2 min later. Storage modulus (G′) and loss modulus (G″) was monitored as a function of time under oscillation frequency sweep (from 1 to 22 Hz) at constant temperature (23 °C). The gel point was achieved when tan d = G″/G′ became independent on frequency. Each sample was measured in triplicate.

### 2.7. Viscoelastic Moduli and Breaking Strength

The viscoelastic moduli (storage modulus G’ and loss modulus G″) and the breaking strength (σ) were obtained using a HAAKE MARS rheometer (Thermo Scientific) using plate geometry (PP20Ti, titanium plateau of 20 mm-diameter). Liquid hydrogel precursor solutions were injected into the well of 12 well plates immediately after mixing, stored under a humid atmosphere, and the measurements started 14 days later (full crosslinking of the network). Storage modulus (G′), loss modulus (G″) and breaking strength (σ) were monitored as a function of strain (from 0.1 to 3000 Pa) at constant frequency and temperature (1 Hz and 23 °C). Each sample was measured in triplicate.

### 2.8. Adhesion

The adhesion of the hydrogels was evaluated via a traction test using a TAHD plus device. First, the hydrogel was molded between two layers of the desired substrate (e.g., bone or cartilage).

**Figure 1:**
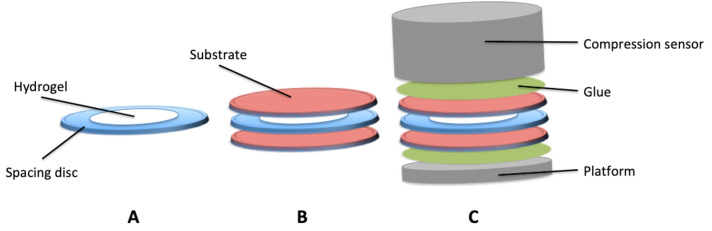
Schematic of the sample preparation for adhesion force measurements. (**A**,**B**) represent the sample preparation with spacing disc well filed with the hydrogel (**A**) and its adhesion onto the desired substrate (**B**). (**C**) Represents the setting of the sample into the TAHDplus measuring device.

To do so, a spacing disc was first prepared with 6 mm external diameter, 4 mm internal diameter and 3/10 mm-thickness (Figure 1A). This spacing disc will be used to have a repeatable thickness of the studied hydrogel.

Then, the samples were prepared as follows: first, a disc of the desired substrate was put into a well of a 12 well plate. Then the spacing disc was put onto the substrate, and the 4 mm well was filled with the desired hydrogel and covered with a second disc of the substrate (Figure 1B). After 90 min of crosslinking, the wells were filled with PBS, and the samples were left for 24 h at 37 °C.

Once the hydrogel was fully crosslinked, the structure was pasted onto the TAHD plus using cyanoacrylate super glue to anchor each faces to the device (Figure 1C). The traction test was performed at a rate of 0.1 mm·s^−1^, and the force was monitored (N).

### 2.9. Cell Investigations

Human adipose stromal cells (hASC) and human chondrosarcoma cell line (SW1353) were cultured in a 5% CO_2_ incubator at 37 °C in Dulbecco’s modified Eagle’s medium (DMEM) (Invitrogen Corp., Villebon sur Yvette, France). Culture media was supplemented with 10% fetal bovine serum, 1% penicillin/streptomycin and 1% L-glutamine and changed each 2–3 days. 

#### 2.9.1. Cell Viability in 2D

SW1353 cells were allowed to attach to 24-well plates at a final density of 10,000 cells per cm^2^. After 24 h, the culture medium was removed, and 500 µL/well of precursor solution of pure Si-HPMC or SI-HPMC/Si-chito were added. The hydrogel samples were incubated at 37 °C for 1 h before adding 1 mL of culture medium. As a positive control, cells were cultured in the absence of Si-HPMC hydrogel. As a negative control, cells were cultured in the presence of actinomycin-D (5 µg mL^−1^), which is a transcription inhibitor. After 1, 2, 3 and 7 days of culture, the hydrogels and culture medium were removed by aspiration. Trypan blue exclusion dye assays were performed to count the cells. In order to evaluate their mitochondrial activity, the methyl tetrazolium salt (MTS) (Promega, Madison, WI, USA) test was performed by adding the MTS solution to each well for 1 h. The optical density of the formazan dye was measured in a spectrophotometer at 490 nm. Each condition was tested in quadruplicate.

#### 2.9.2. Cell Viability in 3D/Cell Adhesion

3D culture cell viability was assessed by using LIVE/DEAD Assay Kits (Invitrogen, Villebon sur Yvette, France) along with confocal image analysis. hASC cells were dispersed in the hydrogel precursor solutions within 5 min following their preparation (thus before gelification) at a final concentration of 1,000,000 cells per mL of hydrogel. Five hundred microliter of each mixture was molded into 24-wells plates and incubated at 37 °C for 1 h to allow the gelification of the hydrogels. Afterward, 1 mL of DMEM medium was added per well, and the samples were incubated for 1, 2, 3, and 7 days before the Live/Dead assays. Actinomycin-D treatment (5 µg mL^−1^) was used as an internal control for cell death. In each well, the culture medium was replaced by 200 µL of a solution containing 2.5 µL of DMEM medium supplemented with 0.25 µL of calcein-AM and 5 µL of ethidium homodimer-1. After 45 min the dye mixture was removed, and the hydrogels were intensively rinsed with phosphate-buffered saline solution before being observed with a confocal laser scanning microscope (Nikon D-eclipse C1) equipped with an argon/krypton laser. Each condition was tested in quadruplicate, and for each sample, two random positions (x, y, z) were chosen within the hydrogel, and a series of images were recorded starting from these positions along the *z*-axis. The 210 images obtained per sample were analyzed with a quantimeter Q550 (Leica Microsystems, Wetzlar, Germany).

### 2.10. In Vivo Experiment

#### 2.10.1. Implants Preparation

Human nasal chondrocytes hNC was used in this experiment. As previously described [17,18], hNC were isolated from human nasal cartilage from surgical waste. Nasal cartilage was cut into small slices and digested at 37 °C with 0.05% hyaluronidase in HBSS for 10 min, then with 0.2% trypsin for 15 min and with 0.2% type II collagenase for 30 min. Finally, slices were digested overnight at 37 °C in 0.03% collagenase in DMEM. The suspended hNC were cultured in DMEM Glutamax supplemented with 10% FCS, 1% penicillin/streptomycin (control medium). Cells were maintained at 37 °C in a humidified atmosphere of 5% CO_2,_ and the culture medium was changed every 2–3 days.

#### 2.10.2. Subcutaneous Implantation

Four swiss nude female mice (seven weeks old) were used for the in vivo study (Charles River Laboratory, Saint germain nuelles, France). All animals were treated in accordance with the Nantes University of Medical Animal Care Guidelines. Four experimental conditions were designed. Two control groups were designed: Si-HPMC was injected alone as negative control and Si-HPMC + hNC as a positive control. Then, 2 experimental groups were designed: Si-HPMC/Si-chito alone and Si-HPMC/Si-chito + hNC. Four samples per mice were implanted subcutaneously in the back along each side of the mice dorsum. Animals were under general anesthesia using isoflurane gas (Halothane, Baxter, Glattpark, Switzerland), and the implantations were performed under aseptic conditions. After 6 weeks, mice were anesthetized and euthanized. Hydrogels were individually dissected and removed from the subcutaneous site. Histological analyses were performed.

#### 2.10.3. Histological Experiments

All histological samples were fixed in 10% formalin and embedded in paraffin. Embedded samples were sectioned (5 µm-thick). Thereafter paraffin sections were deparaffinized using toluene, rehydrated through a graded series of ethanol and rinsed in distilled water. Tissue sections were stained with hematoxylin–eosin-safran (HES), Alcian blue (AB) and Masson’s trichrome (MT). Sections were then visualized using a light microscope (Zeiss Axioplan 2, Göttingen, Germany). HES stains the nucleus in purple, the cytoplasm in pink, collagen fibers in orange, and AB reveals the presence of glycosaminoglycans (GAGs) in blue. Finally, MT stains the nucleus in blue/black, the cytoplasm in pink/red, hematins and keratin in bright red and collagen in green.

### 2.11. Statistics

Results are expressed as mean ± SD of replicate determinations. Comparative studies of means were performed by using two-way ANOVA with minimal statistical significance at *p* < 0.05.

## 3. Results

### 3.1. Chitosan Functionalization

The functionalization of the chitosan backbone was developed through the modification of several chemical parameters. As summarized in Table 1, the key parameters were the control of the pH, the quantity of IPTS and the duration of the reaction. First, the effectiveness of the functionalization was monitored by semi-quantitative SEM-EDX measurements of the silicon within the backbone (Figure S1 supplementary information). The results show the drastic influence of the pH maintenance on the absence of grafting. Indeed, silane groups were effectively grafted onto the chitosan backbone with no control of the pH (reaching a pH value of 5.5 at the final reaction time) while no grafting could be observed when the pH is maintained at pH 3 (pH of chitosan solubilization). At the same time, the influence of the quantity of IPTS was evaluated by adding various quantities (Table 1). As expected, when the quantity of IPTS increased, the efficiency of the grafting increased accordingly. In the same manner, when the duration of the reaction increased, the efficiency of the functionalization increased.

In a second step, the ability of the functionalized chitosan to form a hydrogel was evaluated. The different steps of the formulation (solubilization and hydrogel formation of chitosan alone or mixed with Si-HPMC copolymer) were monitored and are reported in Table 1. As observed in Table 1, because of the non-efficiency of the functionalization, none of the conditions with pH control were able neither to be solubilized nor to form a hydrogel. On the other hand, when the pH was not controlled, the functionalization was effective, and the presence of silanol groups onto the chitosan backbone allows it to be soluble in strongly basic conditions (pH 12.8) after a minimum of 3H of reaction time. Once solubilized (alone or with Si-HPMC), the pH was decreased to 7.4 with ABS, and the ability of silanized chitosan (Si-chito) to form a hydrogel was observed. It appeared that Si-chito alone could form hydrogels at an early time, but syneresis reduced their volume leading to the breaking of the hydrogels. As observed in Table 1, even with Si-HPMC/Si-chito formulation, syneresis could be observed at higher functionalization rates. These preliminary investigations allowed us to select the proper synthesis and formulation conditions: 1 equivalent of IPTS with no pH control for 3H followed by its formulation in association with Si-HPMC copolymer. The selected silanized chitosan was characterized by ICP-ES, and the degree of substitution was calculated as 13% (determination of the DS by ICP-ES, supplementary information).

### 3.2. Rheology

**Figure 2:**
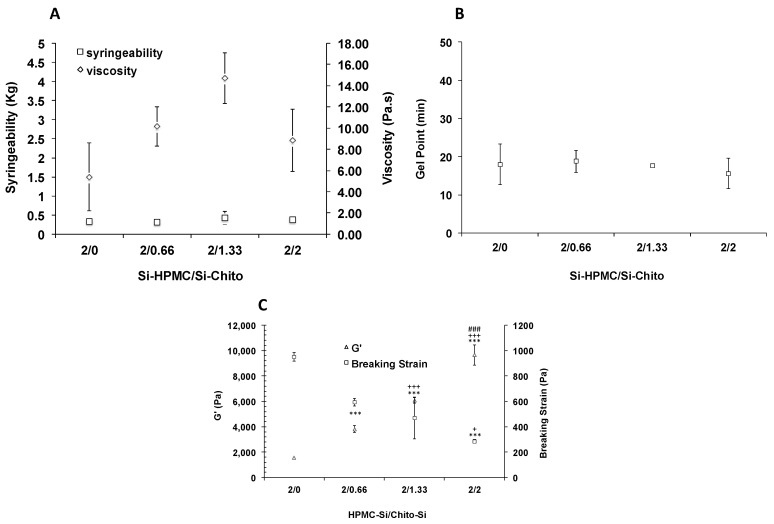
(**A**) Graph representing the injectability (normal force required to eject the solution out of the syringe with a needle (square)) and the viscosity of the silanized chitosan and silanized HPMC mixed solution (diamond). (**B**) Graph representing the gel point (normal force required to eject the solution out of the syringe with a needle (square) and without a needle. (**C**) Graph representing the combined evolution of the storage modulus (G′, triangle) and the breaking strain (σ, square) with an increasing concentration of Si-chitosan. *** *p* < 0.001 compared to the 2/0 condition. ^+^ *p* < 0.05 compared to the 2/0.66 condition. ^+++^ *p* < 0.001 compared to the 2/0.66 condition. ^###^ *p* < 0.001 compared to the 2/1.33 condition.

The selected formulation was then characterized to determine their potential usefulness as hydrogel for tissue engineering. Throughout the physicochemical characterizations, we evaluated and selected the right formulation (in terms of concentration) for biological investigation. First, we measured the viscosity of the solutions. As reported in Figure 2A the viscosity was slightly, but not significantly increased when Si-chito was added as compared with Si-HPMC alone.

Usually, the viscosity profile of solutions can be quite informative of the injectability behavior. Nevertheless, the syringeability of the Si-HPMC/Si-chito solutions was monitored. 50 to 70 N is the maximum force that can be applied by hand [32]. As expected, the syringeability was slightly but not drastically increased when Si-chito was added (Figure 2A).

The gel point, the time point at which the solution becomes a solid, was determine by monitoring the Tan d as a function of time. The time points are reported in Figure 2B, and results showed that there was no influence of the Si-chito concentration on the gel point with a mean value of about 20 min.

### 3.3. Viscoelastic Properties

After evaluating the rheological properties of our hydrogels, we investigated the mechanical behavior to determine either the Si-HPMC/Si-chito could withstand the mechanical force in vivo or not. To do so, the storage modulus (G′) and the breaking strength (σ) at the end of the crosslinking process were measured. The results, plotted in Figure 2C, showed a significant increase of 7 times of G′ when the concentration of Si-chito increase. In the meantime, the breaking strength, representing the fragility of the material, drastically decreased with a diminution of 5 times from 950 to 285 Pa.

### 3.4. Tissue Adhesion

**Figure 3:**
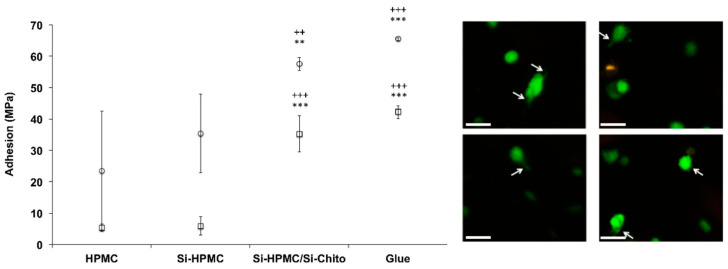
Tissue and cell adhesion. **Left:** graph representing the adhesion of the Si-HPMC/Si-chitosan hydrogel on cartilage (square) and bone (round) surfaces. HPMC was used as negative control and Glue as a positive control. **Right:** confocal micrograph showing the cell interactions after 7 days of cell growth in 3D in Si-HPMC/Si-chitosan hydrogel (2/2, % wt/v). Casein staining shows cells in green. White arrows show cells cytoplasmic prolongments in 3D. Scale bar: 100 µm ** *p* < 0.01 compared to the HPMC condition *** *p* < 0.001 compared to the HPMC condition ^++^ *p* < 0.01 compared to the Si-HPMC condition ^+++^ *p* < 0.001 compared to the Si-HPMC condition.

As preclinical in vitro evaluation and to complete the mechanical measurements, it was critical to determine the adhesion of the hydrogels to the host tissues. The evaluation of the adhesiveness was evaluated onto freshly harvested cartilage and bone using Si-HPMC/Si-chito (2%/2%, final concentration) compared to Si-HPMC alone, HPMC as negative control and cyanoacrylate super glue^®^ as a positive control. The results (Figure 3) showed a significant increase in the adhesion of Si-HPMC/Si-chito (2/2) on the bone. The adhesion on the cartilage surface reached an even higher value with an increase of 5 times compared to Si-HPMC alone and no significant difference with cyanoacrylate super glue^®^.

Moreover, the confocal analysis allowed us to have a look at the shape of the embedded cells. While the hASC usually showed a round spherical shape (data not are shown), specific of the non-adherent cell, after 7 days of culturing within Si-HPMC/Si-chito hydrogels, they displayed a stretching of their shape with filaments (Figure 3), leading to an attachment process.

### 3.5. Cell Investigations

The aim of the cellular studies was to evaluate the cytotoxicity of our mixed hydrogels in 2D (hydrogel above the cell layer) and in 3D (cell proliferation within the hydrogel).

Throughout the physicochemical characterizations, we have selected the best condition to synthesize and formulate the Si-chito: 1 equivalent of IPTS with no pH control for 3H followed by its formulation in association with Si-HPMC as copolymer.

**Figure 4:**
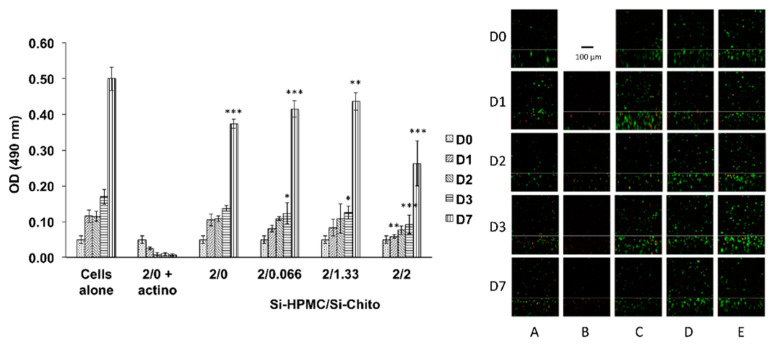
**Left:** graph representing SW1353 2D cell metabolism (cell growth) with an increasing Si-HPMC concentration over a period of time of 7 days. **Right:** confocal micrograph representing the 3D cell viability with an increasing Si-chito (Si-HPMC/Si-chito: A: 2%/0%; B: 2%/0% + actinomycin; C: 2%/0.66%; D: 2%/1.33 and E: 2%/2%) concentration over a period of time of 7 days (D0, D1, D2, D3 and D7) using LIVE/DEAD staining kit labeling living cells in green and dead cells in red. Si-HPMC alone was used as positive control and actinomycin as control of cell death. Scale bar: 100 µm. * *p* < 0.05 compared to the cells alone condition ** *p* < 0.01 compared to the cells alone condition *** *p* < 0.001 compared to the cells alone condition

The selected Si-chito was formulated with an increasing ratio, and the cell metabolism was monitored using MTS assay on days 1, 2, 3 and 7. The results were plotted in Figure 4 and demonstrated that there was no effect of the hydrogels on the cellular metabolism that are linked to viability and cellular proliferation over time. Following the 2D cytotoxicity assay evaluation, 3D cell viability was studied (Figure 4). The confocal micrographs show good viability of the hASC cultured within the Si-HPMC/Si-chito hydrogels regardless of the concentration of Si-chito. In the meantime, the cells cultured in Si-HPMC alone as positive control show good viability, and the one cultured in the presence of actinomycin-D died (negative control).

### 3.6. In Vivo Experiment

**Figure 5:**
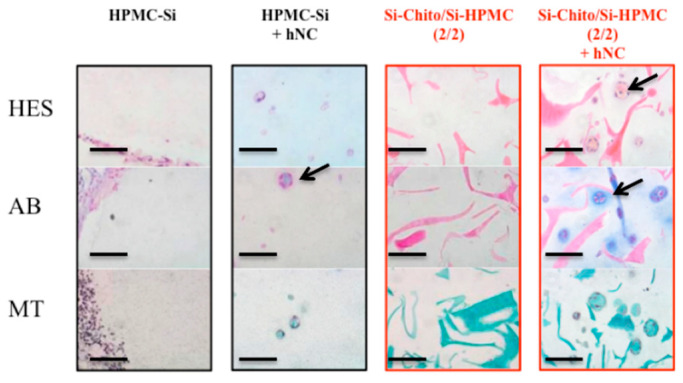
hCN were cultured and implanted with the Si-HPMC/Si-chitosan (2/2, % wt/v) hydrogel into subcutaneous pockets of nude mice (right column) and in Si-HPMC hydrogel as a positive control (2nd column). Si-HPMC (2% wt/v) and Si-HPMC/Si-chitosan (2/2, % wt/v) hydrogel without cells were used as negative control. 1 million cells/mL were associated with hydrogel. Hematoxylin–eosin-safran (HES) staining, (1st row) Alcian blue (AB) staining (2nd row) and Masson’s trichrome (MT) staining (3rd row) were performed for all conditions. Scale bar: 100 µm.

Towards the utilization of Si-HPMC/Si-chito hydrogels for tissue engineering purposes, the next step was to investigate if they may be able to support the chondrogenic activity of chondrocytes by detecting the presence of chondrogenic extracellular matrix components. Consequently, we performed an in vivo implantation experiment in nude mice subcutis. After harvest, isolation and culture, hNC (1 to 5 × 10^6^ cells/mL) were mixed with Si-HPMC or Si-HPMC/Si-chito hydrogels and injected in subcutis for 6 weeks. Histological staining demonstrated that the conditions without chondrocytes (Si-HPMC and SI-HPMC/Si-chito) showed no cells. However, some colorations were observed within the graft as the chitosan interacted with the histological staining molecules. These staining can suggest that both silated polysaccharides are not fully miscible and areas with more concentrated Silated chitosan seem to be visible by the 3 stainings. Figure 5 shows that hNC associated with Si-HPMC and Si-HPMC/Si-chito hydrogels are organized in nodules revealed in HES, AB and MT staining. HES staining also indicated the presence of mature chondrocytes (black arrows). Cells in the nodules secreted GAGs revealed in blue with AB staining, and collagens evidenced in green with MT staining (black arrows). Moreover, an increase in the number of nodules can be observed in the Si-HPMC/Si-chito hydrogel conditions.

## 4. Discussion

The primary goal of this study was to develop a self-setting and injectable hydrogel based on the use of chitosan. This polymer was selected not only for its known compatibility and biodegradability but also because of its reported biological properties (efficient delivery, no cytotoxicity, mucosa adhesiveness) [33,34,35,36].

The strategy of the self-setting formulation of the hydrogel was based on the silane chemistry. This process was selected with regard to previous work of the laboratory dealing with Silated HPMC and in the objective to get functionalized polymers using the same chemistry for the development of tunable hydrogels.

Si-HPMC allows in situ forming hydrogels based on inorganic–organic chemistry, leveraging the self-condensation of grafted silanolate moieties (Si-O-Na^+^) into siloxanes (Si-O-Si) under physiological conditions [28]. This original inorganic crosslinking mechanism allows hybrid covalent hydrogels [37] that simply occurs upon pH neutralization prior to injection, without any detrimental influence on living tissues [38]. In bone, it has been reported that the Si-HPMC hydrogel used alone acts as a low degradable barrier [15,39,40], and in cartilage, it has been shown to have no interactions with cells [16]. For biomaterial substitutes and scaffold for tissue engineering, we needed variable stiffness and cell or tissue adhesive properties self-setting injectable hydrogel for different targeted tissue regenerations.

Nevertheless, the chemistry for the functionalization of the chitosan had to be defined and optimized. Toward that objective, we first screened the chemical parameters of the grafting of silane moieties onto the chitosan backbone. As reported in Table 1, 3 main parameters were evaluated (pH, silane concentration and the reaction time). The proper conditions of synthesis were selected based on the functionalization efficiency and the ability of the silated chitosan to form a hydrogel. Briefly, to form a hydrogel, the silated chitosan was first dissolved in a strongly basic medium (NaOHaq, pH 12.5) and then neutralized using an acid buffer to reach pH 7. Altogether, these criteria allowed us to select the synthesis without control of the pH, 1 equivalent and onwards of (3-Isocyanatopropyl)triethoxysilane and a minimum of 3 h of reaction time.

Following the evaluation of the silated chitosan to form a hydrogel, we further characterized the selected functionalized polymers to determine the optimal formulation for skeletal tissue engineering purposes. Toward that goal, we first formulate Si-chito alone. The pH based setting of the hydrogel allowed us to form hydrogels using Si-chito as the only polymer network. However, as reported in the literature [41,42,43], the protocol used induced high syneresis properties of the hydrogels with a drastic contraction leading to the destruction of the samples. Therefore, we added Si-HPMC as copolymer to maintain the architectural shape of the hydrogels. Consequently, we developed the formulations of the Si-HPMC/Si-chito composite hydrogels incorporating several ratios of Si-chito. The flow rheology measurements have shown no modification of both the viscosity and the injectability, regardless of the quantity of Si-chito. Following the flow rheology measurements, the viscoelastic and mechanical properties of the hydrogels were evaluated. The storage (elastic) modulus G’ provides information about the elasticity or energy stored in the material during deformation, whereas the loss (viscous) modulus G″ describes the viscous character or energy dissipated on heating. The ratio between the viscous and elastic moduli is expressed by the loss tangent (tan d = G″/G′), where d is the phase angle. The loss tangent is a measurement of the ratio of energy lost against energy stored in a cyclic deformation. Compared to previous studies using Si-HPMC alone [28,29,30] or Si-HPMC formulated with reinforcement [9,13,44], adding Si-chito reaches to higher viscoelastic modulus with a 9-fold increase compared to a 4-fold increase with XLG laponites or blended with others marine polysaccharides [16]. However, as expected, the increase of the rigidity of the hydrogels goes with a decrease of the breaking stress making the final construct five times more fragile compared to the hydrogels without Si-chito.

Human adipose stromal cells have widely been used in skeletal tissue engineering investigations. Their use demonstrated good cell viability with no statistical differences up to 2% of Si-chito in 2D cell viability. In addition, in 3D cell cultures, cells were found living (Figure 4) within the hydrogel for seven days at the Si-chito concentrations up to 2%. Moreover, according to the adhesion properties of the chitosan, confocal micrographs show that the 3D cell culturing induces the loss of the round shape of the cells and the appearance of filaments (Figure 3), which may suggest the attachment of the cells onto the hydrogel network.

Prior to the preclinical in vivo investigations of our hydrogels, their adherence to freshly harvested tissue surfaces was evaluated. The traction tests clearly demonstrated the increase of the adhesion of the hydrogels on cartilage and bone surface with the same adhesion force than with cyanoacrylate super glue (positive control). The adhesion onto the surface of the targeted tissue is essential for tissue engineering. Indeed, the maintenance on the site will give the time required for the cellularized hydrogel to produce the extracellular matrix molecules and potentially heal the damaged tissue.

In this paper, we focus on the use of chondrocytes to evaluate the potential use of our hydrogels for skeletal tissue engineering. The ideal biomaterial should not only allow for the maintenance of the chondrocyte differentiation but should also enable their transplantation via a mini-invasive surgical protocol in vivo. Consequently, to decipher whether Si-HPMC/Si-chito hydrogels could represent this ideal scaffold, we investigated its ability to support the transplantation of chondrogenic cells and the formation of cartilage tissue in vivo. To address this issue, we focused our attention on the use of cells exhibiting a reliable and robust chondrogenic potential such as hNC [17,18]. Interestingly, after six weeks in vivo, hNC were positively stained after, respectively, Alcian blue and Masson Trichrome histology staining confirming that the entrapped hNC could still produce an extracellular matrix containing GAG and collagen. Moreover, the histological staining has shown the presence of aggregates within the Si-HPMC/Si-chito networks demonstrating the nonhomogeneous structure of the hydrogels. In addition to the properties of the chitosan, these aggregates may be responsible for the cell adhesion properties of the hASC within the hydrogels.

## 5. Conclusions

In conclusion, we have developed the synthesis of silanized chitosan, which can be formulated with Si-HPMC to obtain a stiffer hydrogel showing a stronger adhesion onto the surface of tissues. This self-setting hydrogel showed good cytocompatibility and can support cell viability and extracellular matrix secretion in a subcutaneous pocket in nude mice. The efficiency in a relevant animal model for cartilage or osteochondral defects regeneration will now be conducted as a preclinical investigation.

## Figures and Tables

**Table 1:** Table summarizing the characterizations of the silanized chitosan, e.g., effectiveness of the silanization (determined via EDX (Electron Difraction X-ray) analyses), solubility in 0.2 M NaOHaq and the ability of the silanized chitosan to form a hydrogel (alone or mixed with Si-HPMC).

**Relative Quantity Chitosan/ICPTS**			**1/1**					**1/1.5**				
Reaction time (H)			1	2	3	4	5	1	2	3	4	5
Uncontroled pH	grafting efficiency		+	+	++	++	++	+	+	++	++	++
	Solubility		-	-	+	+	+	-	-	+	+	+
	Hydrogel formation	alone	-	-	+ (syneresis)	+ (syneresis)	+ (syneresis)	-	-	+ (syneresis)	+ (syneresis)	+ (syneresis)
		mixt with Si-HPMC	-	-	+	+	+	-	-	+	+	+ (syneresis)
Controled pH	grafting efficiency		-	-	-	-	-	-	-	-	-	-
	Solubility		-	-	-	-	-	-	-	-	-	-
	Hydrogel formation	alone	-	-	-	-	-	-	-	-	-	-
		mixt with Si-HPMC	-	-	-	-	-	-	-	-	-	-
**Relative Quantity Chitosan/ICPT**S			**1/2**					**1/2.5**				
Reaction time (H)			1	2	3	4	5	1	2	3	4	5
Uncontroled pH	grafting efficiency		+	+	++	++	++	+	+	++	++	++
	Solubility		-	-	+	+	+	-	-	+	+	+
	Hydrogel formation	alone	-	-	+ (syneresis)	+ (syneresis)	+ (syneresis)	-	-	+ (syneresis)	+ (syneresis)	+ (syneresis)
		mixt with Si-HPMC	-	-	+	+ (syneresis)	+ (syneresis)	-	-	+ (syneresis)	+ (syneresis)	+ (syneresis)
Controled pH	grafting efficiency		-	-	-	-	-	-	-	-	-	-
	Solubility		-	-	-	-	-	-	-	-	-	-
	Hydrogel formation	alone	-	-	-	-	-	-	-	-	-	-
		mixt with Si-HPMC	-	-	-	-	-	-	-	-	-	-

- unable + able or effective grafting ++ higher grafting compared to +.

## Supplementary Materials

The following are available online at https://www.mdpi.com/2073-4360/12/12/2823/s1, Figure S1: SEM-EDX micrograph showing the detected elements of the polymer. It demonstrates the presence of Si within the backbone of chitosan. The Si grafting was monitored by SEM-EDX measurements of all samples. Characterization of the DS by ICP-AES, Figure S2: hCN were cultured and implanted with the Si-HPMC/Si-chitosan (2/2, %wt/v) hydrogel into subcutaneous pockets of nude mice. (Right column) and in Si-HPMC hydrogel as positive control (2nd column). Si-HPMC (2% wt/v) and Si-HPMC/Si-chitosan (2/2, %wt/v) hydrogel without cells were used as negative control. 1 million cells / mL were associated with hydrogel. HES staining, (1st row) AB staining (2nd row) and MT staining (3rd row) were performed for all conditions. Picture was taken at 10× magnification (A) and 20× magnification (B).

## References

[1] Flégeau K., Pace R., Gautier H., Rethore G., Guicheux J., Le Visage C., Weiss P. (2017). Toward the development of biomimetic injectable and macroporous biohydrogels for regenerative medicine. Adv. Colloid Interface Sci..

[2] Chun H.J., Park K., Kim C., Khang G. (2018). Novel Biomaterials for Regenerative Medicine.

[3] Engler A.J., Sen S., Sweeney H.L., Discher D.E. (2006). Matrix Elasticity Directs Stem Cell Lineage Specification. Cell.

[4] Badylak S.F. (2007). The extracellular matrix as a biologic scaffold material. Biomaterials.

[5] McCall J.D., Luoma J.E., Anseth K.S. (2012). Covalently tethered transforming growth factor beta in PEG hydrogels promotes chondrogenic differentiation of encapsulated human mesenchymal stem cells. Drug Deliv. Transl. Res..

[6] Edgar L., McNamara K., Wong T., Tamburrini R., Katari R., Orlando G. (2016). Heterogeneity of Scaffold Biomaterials in Tissue Engineering. Materials.

[7] Dornish M., Kaplan D., Skaugrud O. (2001). Bioartificial Organs III: Tissue Sourcing, Immunoisolation, and Clinical Trials. Ann. N. Y. Acad. Sci..

[8] Guan X., Avci-Adali M., Alarçin E., Cheng H., Kashaf S.S., Li Y., Chawla A., Jang H.L., Khademhosseini A. (2017). Development of hydrogels for regenerative engineering. Biotechnol. J..

[9] Boyer C., Figueiredo L., Pace R., Lesoeur J., Rouillon T., Le Visage C., Tassin J.-F., Weiss P., Guicheux J., Rethore G. (2018). Laponite nanoparticle-associated silated hydroxypropylmethyl cellulose as an injectable reinforced interpenetrating network hydrogel for cartilage tissue engineering. Acta Biomater..

[10] Boyer C., Réthoré G., Weiss P., D’Arros C., Lesoeur J., Vinatier C., Halgand B., Geffroy O., Fusellier M., Vaillant G. (2020). A Self-Setting Hydrogel of Silylated Chitosan and Cellulose for the Repair of Osteochondral Defects: From in vitro Characterization to Preclinical Evaluation in Dogs. Front. Bioeng. Biotechnol..

[11] Figueiredo L., Pace R., D’Arros C., Réthoré G., Guicheux J., Le Visage C., Weiss P. (2018). Assessing glucose and oxygen diffusion in hydrogels for the rational design of 3D stem cell scaffolds in regenerative medicine. J. Tissue Eng. Regen. Med..

[12] Moussa L., Demarquay C., Réthoré G., Benadjaoud M.A., Siñeriz F., Pattappa G., Guicheux J., Weiss P., Barritault D., Mathieu N. (2019). Heparan Sulfate Mimetics: A New Way to Optimize Therapeutic Effects of Hydrogel-Embedded Mesenchymal Stromal Cells in Colonic Radiation-Induced Damage. Sci. Rep..

[13] Xie F., Boyer C., Gaborit V., Rouillon T., Guicheux J., Tassin J.-F., Geoffroy V., Rethore G., Weiss P. (2018). A Cellulose/Laponite Interpenetrated Polymer Network (IPN) Hydrogel: Controllable Double-Network Structure with High Modulus. Polymers.

[14] Song R., Murphy M., Li C., Ting K., Soo C., Zheng Z. (2018). Current development of biodegradable polymeric materials for biomedical applications. Drug Des. Dev. Ther..

[15] Fellah B.H., Weiss P., Gauthier O., Rouillon T., Pilet P., Daculsi G., Layrolle P. (2006). Bone repair using a new injectable self-crosslinkable bone substitute. J. Orthop. Res..

[16] Rederstorff E., Weiss P., Sourice S., Pilet P., Xie F., Sinquin C., Colliec-Jouault S., Guicheux J., Laïb S. (2011). An in vitro study of two GAG-like marine polysaccharides incorporated into injectable hydrogels for bone and cartilage tissue engineering. Acta Biomater..

[17] Vinatier C., Magne D., Moreau A., Gauthier O., Malard O., Vignes-Colombeix C., Daculsi G., Weiss P., Guicheux J. (2007). Engineering cartilage with human nasal chondrocytes and a silanized hydroxypropyl methylcellulose hydrogel. J. Biomed. Mater. Res. Part A.

[18] Vinatier C., Gauthier O., Fatimi A., Merceron C., Masson M., Moreau A., Moreau F., Fellah B., Weiss P., Guicheux J. (2009). An injectable cellulose-based hydrogel for the transfer of autologous nasal chondrocytes in articular cartilage defects. Biotechnol. Bioeng..

[19] Croisier F., Jérôme C. (2013). Chitosan-based biomaterials for tissue engineering. Eur. Polym. J..

[20] Durkin C.A., Mock T., Armbrust E.V. (2009). Chitin in Diatoms and Its Association with the Cell Wall. Eukaryot. Cell.

[21] Agboh O.C., Qin Y. (1997). Chitin and chitosan fibers. Polym. Adv. Technol..

[22] Kim S.J., Park S.J., Kim S.I. (2003). Swelling behavior of interpenetrating polymer network hydrogels composed of poly(vinyl alcohol) and chitosan. React. Funct. Polym..

[23] Ong S.-Y., Wu J., Moochhala S.M., Tan M.-H., Lu J. (2008). Development of a chitosan-based wound dressing with improved hemostatic and antimicrobial properties. Biomaterials.

[24] Sudarshan N.R., Hoover D.G., Knorr D. (1992). Antibacterial action of chitosan. Food Biotechnol..

[25] Khor E., Lim L.Y. (2003). Implantable applications of chitin and chitosan. Biomaterials.

[26] Shahidi F., Abuzaytoun R. (2005). Chitin, Chitosan, and Co-Products: Chemistry, Production, Applications, and Health Effects.

[27] Ahmadi F., Oveisi Z., Mohammadi-Samani S., Amoozgar Z. (2015). Chitosan based hydrogels: Characteristics and pharmaceutical applications. Res. Pharm. Sci..

[28] Bourges X., Weiss P., Daculsi G., Legeay G. (2002). Synthesis and general properties of silated-hydroxypropyl methylcellulose in prospect of biomedical use. Adv. Colloid Interface Sci..

[29] Fatimi A., Tassin J.F., Quillard S., Axelos M.A., Weiss P. (2008). The rheological properties of silated hydroxypropylmethylcellulose tissue engineering matrices. Biomaterials.

[30] Fatimi A., Tassin J.-F., Turczyn R., Axelos M.A., Weiss P. (2009). Gelation studies of a cellulose-based biohydrogel: The influence of pH, temperature and sterilization. Acta Biomater..

[31] Cross M.M. (1965). Rheology of non-Newtonian fluids: A new flow equation for pseudoplastic systems. J. Colloid Sci..

[32] Vo A., Doumit M., Rockwell G. (2016). The Biomechanics and Optimization of the Needle-Syringe System for Injecting Triamcinolone Acetonide into Keloids. J. Med. Eng..

[33] Dash B.C., Réthoré G., Monaghan M., Fitzgerald K., Gallagher W., Pandit A. (2010). The influence of size and charge of chitosan/polyglutamic acid hollow spheres on cellular internalization, viability and blood compatibility. Biomaterials.

[34] Rethore G., Mathew A., Naik H., Pandit A. (2009). Preparation of Chitosan/Polyglutamic Acid Spheres Based on the Use of Polystyrene Template as a Nonviral Gene Carrier. Tissue Eng. Part C Methods.

[35] Lee K.Y., Mooney D.J. (2001). Hydrogels for Tissue Engineering. Chem. Rev..

[36] Debnath T., Ghosh S., Potlapuvu U.S., Kona L., Kamaraju S.R., Sarkar S., Gaddam S., Chelluri L.K. (2015). Proliferation and Differentiation Potential of Human Adipose-Derived Stem Cells Grown on Chitosan Hydrogel. PLoS ONE.

[37] Montheil T., Echalier C., Martinez J., Subra G., Mehdi A. (2018). Inorganic polymerization: An attractive route to biocompatible hybrid hydrogels. J. Mater. Chem. B.

[38] Trojani C., Boukhechba F., Scimeca J.-C., Vandenbos F., Michiels J.-F., Daculsi G., Boileau P., Weiss P., Carle G.F., Rochet N. (2006). Ectopic bone formation using an injectable biphasic calcium phosphate/Si-HPMC hydrogel composite loaded with undifferentiated bone marrow stromal cells. Biomaterials.

[39] Chichiricco P.M., Riva R., Thomassin J.-M., Lesoeur J., Struillou X., Le Visage C., Jérôme C., Weiss P. (2018). In situ photochemical crosslinking of hydrogel membrane for Guided Tissue Regeneration. Dent. Mater..

[40] Zhang J., Liu W., Gauthier O., Sourice S., Pilet P., Rethore G., Khairoun K., Bouler J.-M., Tancret F., Weiss P. (2016). A simple and effective approach to prepare injectable macroporous calcium phosphate cement for bone repair: Syringe-foaming using a viscous hydrophilic polymeric solution. Acta Biomater..

[41] Duggan E., Waghorne W.E. (2001). Effect of addition of chitosan on rheological properties of acidified milk gels. Trends in Colloid and Interface Science XV.

[42] Moore G.K., Roberts G.A. (1980). Chitosan gels: 1. Study of reaction variables. Int. J. Biol. Macromol..

[43] Ramanathan S., Block L.H. (2001). The use of chitosan gels as matrices for electrically-modulated drug delivery. J. Control. Release.

[44] Buchtová N., Réthoré G., Boyer C., Guicheux J., Rambaud F., Vallé K., Belleville P., Sanchez C., Chauvet O., Weiss P. (2013). Nanocomposite hydrogels for cartilage tissue engineering: Mesoporous silica nanofibers interlinked with siloxane derived polysaccharide. J. Mater. Sci. Mater. Med..

