# Supplementary material for: Silanization of Chitosan and Hydrogel Preparation for Skeletal Tissue Engineering
Source: Polymers (Basel). 2020 Nov 27;12(12):2823. doi: 10.3390/polym12122823 (PMC7761294; doi:10.3390/polym12122823)
Supplement: Supplementary file 1 [file polymers-12-02823-s001.pdf]

polymers

MDPI

Supplementary informations of

Silanization of Chitosan and Hydrogel Preparation for Skeletal Tissue Engineering

Gildas Réthoré [1,2,3], Cécile Boyer [1,2], Kouakou Kouadio [1,2], Amadou Toure [1,2,4], Julie Lesoeur [1,2] Boris Halgand [1,2,3], Fabienne Jordana [1,2,3], Jérôme Guicheux [1,2,3], and Pierre Weiss [1,2,3,*]

1 Université de Nantes, UMR 1229, RMeS, Regenerative Medicine and Skeleton, INSERM, ONIRIS, F-44042 Nantes, France; gildas.rethore@univ-nantes.fr (G.R.); cecile.boyer@univ-nantes.fr (C.B.); kouadiokouakou@yahoo.fr (K.K.); amad_toure@yahoo.fr (A.T.); julie.lesoeur@univ-nantes.fr (J.L.); boris.halgand@univ-nantes.fr (B.H.); fabienne.jordana@univ-nantes.fr (F.J.); jerome.guicheux@univ-nantes.fr (J.G.)
2 Université de Nantes, UFR Odontologie, F-44042 Nantes, France
3 CHU Nantes, PHU4 OTONN, F-44093 Nantes, France
4 Department of Odontology, Faculty of Medicine, Pharmacy and Odontology, University Cheikh Anta DIOP, 12500 Dakar, Senegal
* Correspondence: pierre.weiss@uiv-nantes.fr

Received: 25 October 2020; Accepted: 24 November 2020; Published: 27 November 2020

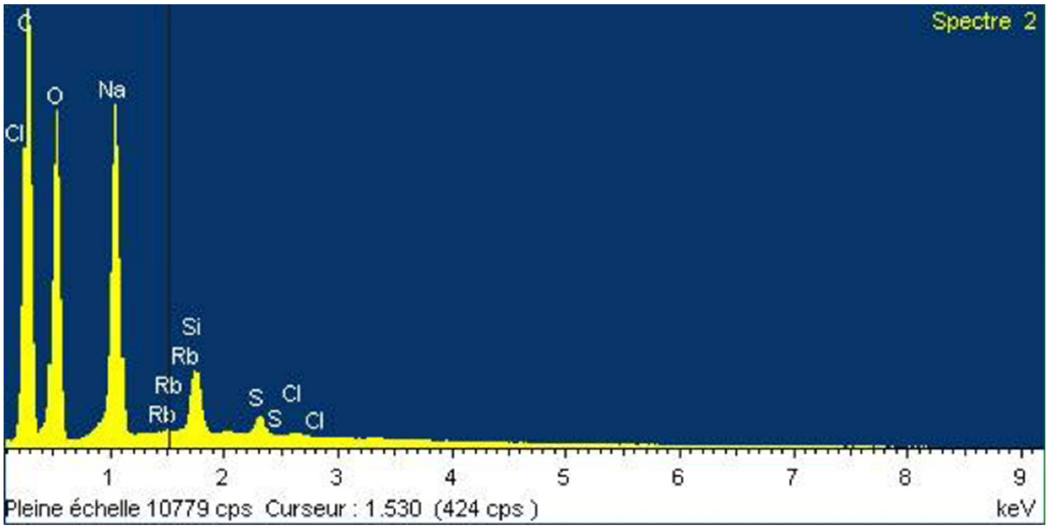

Figure S1. SEM-EDX micrograph showing the detected elements of the polymer. It demonstrates the presence of Si within the backbone of chitosan. The Si grafting was monitored by SEM-EDX measurements of all samples.

Characterization of the DS by ICP-AES

The degree of substitution was determined by induced coupled plasma-atomic emission spectroscopy (ICP-AES). Determination of silicon content was outsourced to the Pole Spectrometry Ocean Brest (PSO, Brest, France), where measurements were performed using an Ultima2, Hobira Jobin Yvon spectrometer. The percentage of APTES grafted to the polymer backbone was calculated using the following equation:

$$DS(\%) = \%_{grafted\ Si} = \frac{\frac{\%Si \times MChito}{MSi}}{\frac{[1 - \%Si \times (MChito - IPTS - MChito)]}{100 \times MSi}} \quad (1)$$

With %Si (w/w), the value obtained by ICP-AES; MChito = 207.4 $g.mol^{-1}$ (80% deacetylation); MSi = 28.08 $g.mol^{-1}$; and MHA-IPTS = 454.76 $g.mol^{-1}$.

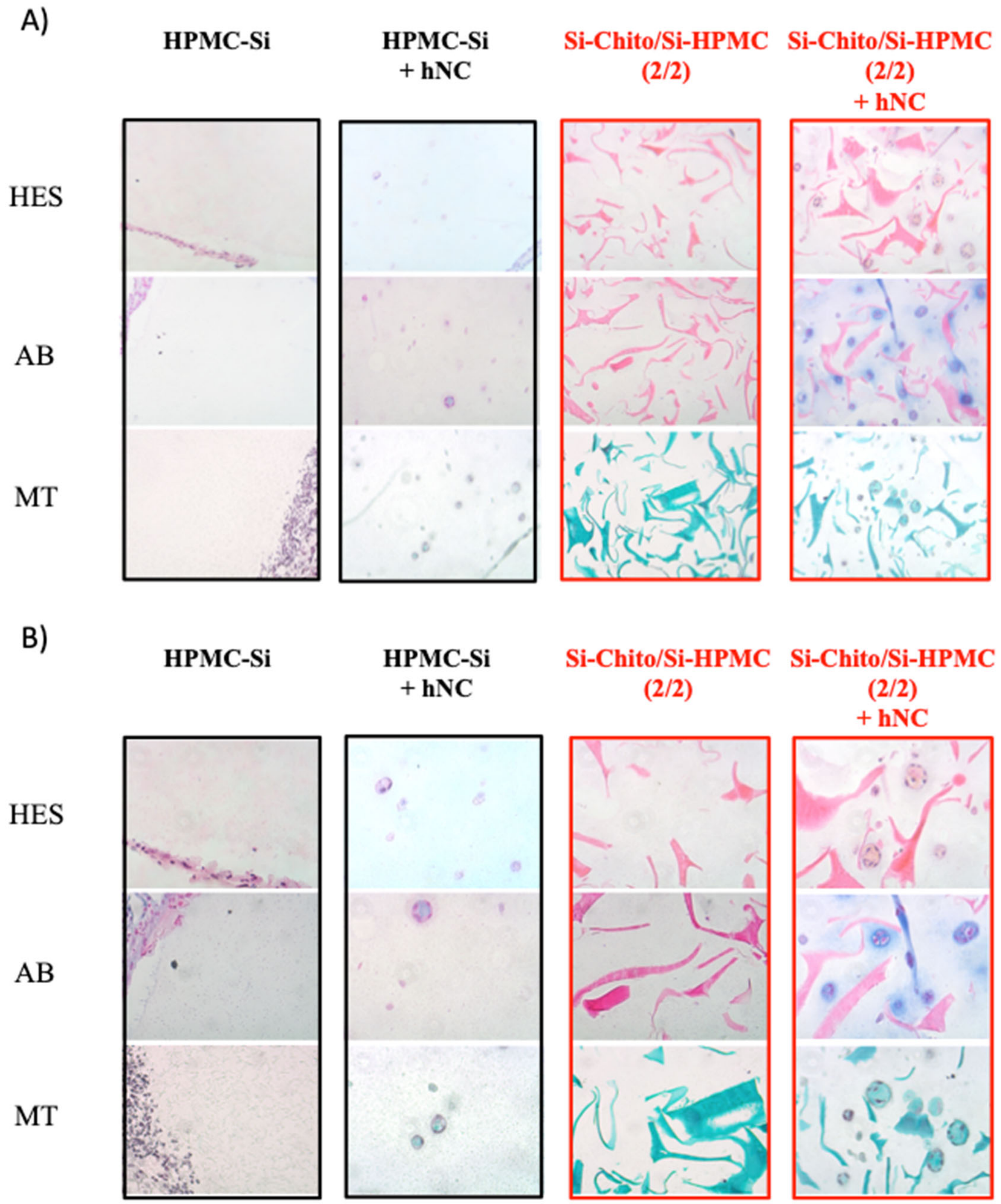

Figure S2. hCN were cultured and implanted with the Si-HPMC/Si-chitosan (2/2, %wt/v) hydrogel into subcutaneous pockets of nude mice. (right column) and in Si-HPMC hydrogel as positive control (2nd column). Si-HPMC (2%, wt/v) and Si-HPMC/Si-chitosan (2/2, %wt/v) hydrogel without cells were used as negative control. 1 million cells / mL were associated with hydrogel. HES staining, (1st row) AB staining (2nd row) and MT staining (3rd row) were performed for all conditions. Picture was taken at 10x magnification (**A**) and 20x magnification (**B**).

Publisher's Note: MDPI stays neutral with regard to jurisdictional claims in published maps and institutional affiliations.

© 2020 by the authors. Licensee MDPI, Basel, Switzerland. This article is an open access article distributed under the terms and conditions of the Creative Commons Attribution (CC BY) license (http://creativecommons.org/licenses/by/4.0/).
